# Effects of feeding ethanol on growth performances, carcass characteristics, and lipid metabolism of finishing Korean cattle (Hanwoo) steers

**DOI:** 10.5713/ajas.18.0853

**Published:** 2019-01-04

**Authors:** Chang Bon Choi, Hana Kwon, Kyung Hyun Hwang, Hyun-Jeong Lee, Jong Yeon Kim

**Affiliations:** 1 Department of Medical Biotechnology, College of Life and Applied Sciences, Yeungnam University, Gyeongsan 38541, Korea; 2 Department of Food and Nutrition, Yeungnam University, Gyeongsan 38541, Korea; 3 Gunwi Livestock Cooperative Federation, Gunwi 39015, Korea; 4 Animal Nutritional Physiology Team, National Institute of Animal Sciences, Wanju 55365, Korea; 5 Department and Faculty of Physiology, College of Medicine, Yeungnam University, Daegu 42415, Korea

**Keywords:** Steers, Ethanol, Steers, Intramuscular Fat, Lipid Metabolism, Non-esterified Fatty Acid, Triglycerides

## Abstract

**Objective:**

The purpose of this study was to determine the effects of feeding ethanol on growth performances, carcass characteristics, and lipid metabolism of finishing Korean cattle (Hanwoo) steers.

**Methods:**

Thirty (30) Hanwoo steers (average 25.1 months of age, body weight 660.1 kg) were assigned to three treatments: control (0% ethanol), E-3 (1.44% ethanol for 3 months), or E-5 (0.72% ethanol for 2 months followed by 1.44% ethanol for 3 months). The animals were allotted by treatment group into six pens and fed concentrate and perennial ryegrass. Ethanol (30%, v/v) was supplemented into drinking water twice a day to meet final concentrations based on average water consumption of finishing Hanwoo steers.

**Results:**

There were no statistical differences among the groups in final body weight, average daily gain, or carcass yield grade indices such as cold carcass weight, fat thickness, and loin area. The marbling score tended (p = 0.228) to increase with the highest score (6.7) in the E-5 group followed by 6.3 and 6.0 in E-3 and control groups, respectively. The appearance frequencies of quality grades of 1^++^ (the best), 1^+^, 1, and 2, were; 30%, 50%, 0%, and 20% for control, 10%, 80%, 10%, and 0% for E-3, and 10%, 80%, 0%, and 10% for E-5 groups, respectively, indicating improvements of quality grades by feeding ethanol. Concentrations of serum glucose tended to decrease whereas those of insulin and non-esterified fatty acid to increase by feeding ethanol (E-3 and E-5; p>0.05).

**Conclusion:**

Feeding ethanol directly into drinking water of finishing Hanwoo steers stimulated lipogenesis in intramuscular adipose tissue (marbling) and thereby improved carcass quality grade. The serum metabolites results supported the hypothesis of lipolysis of existing adipose tissue, such as abdominal fats, and lipogenesis in intramuscular adipocytes.

## INTRODUCTION

Marbling (intramuscular fat) has been shown to play an important role in eating quality of beef by affecting tenderness [1], but marbling regulation varies depending on genetic and environmental backgrounds. Feeding grain, mostly corn, and increasing time on such feed improves the palatability and acceptability of meat, primarily by increasing the amount of intramuscular fat mass (marbling) [2,3].

Production of extensively and highly marbled premium domestic beef is very important to the Korean beef industry in order to provide competitiveness with less expensive and lean imported beef. The main feeding-related features of the Korean cattle (Hanwoo) industry are feeding a high amount of concentrates and rice straw as the sole roughage source [4]. This unique beef cattle feeding program for Korean cattle (Hanwoo) has resulted in highly marbled beef with especially high percentages of oleic acid (~50%), a major monounsaturated fatty acids (MUFA) in beef, leading to a high ratio of MUFA/saturated fatty acids (SFA) of 1.28 [5].

Recently, researchers have been trying to develop new technologies suitable for a more efficient production of highly marbled beef. Selective lipid deposition in meat animals is a relatively new strategy for improving production efficiency while also improving meat quality. However, very little has been reported about the selective regulation of fat deposition into desired depots, such as into intramuscular adipose tissue. Therefore, elucidating preadipocytes and adipocyte biology is vital for altering meat animal carcass composition [3].

However, there are few reference sources fed ethanol directly to steers through drinking water [6] or through mixing in their diets [7], and, in addition, the data in these references are limited to growth performances and carcass characteristics lacking information for changes in lipid metabolism. The other reports related with feeding alcohol to cattle are related to feeding alcohol-fermented diets or by-products of ethanol industries.

After considering the findings in previous reports in the human medicine and animal science fields, we established the hypothesis that ethanol in finishing beef cattle might stimulate lipolysis of pre-existing fat mass, especially abdominal fats, and non-esterified fatty acids (NEFA) released into serum by breakdown of lipids might be used in the synthesis of triglycerides (TG) in intramuscular adipocytes, which would improve the marbling score and, thereby, carcass quality grades. Thus, the purpose of this study was to determine the effects of feeding ethanol directly through drinking water of finishing Hanwoo steers on growth performances, carcass characteristics, and lipid metabolism.

## MATERIALS AND METHODS

### Experimental animals and diets

All experimental procedures for this study were approved by the Institutional Animal Care and Use Committee of Yeungnam University, Korea. Thirty (30) Korean cattle (Hanwoo) steers (25.1±0.3 months of age, 660.1±47.0 kg) in a local farm were assigned to control (0% ethanol), E-3 (fed 1.44% [v/v] ethanol for 3 months until termination of the trial), or E-5 (0.72% ethanol for 2 months followed by 1.44% ethanol for 3 months). The animals were allotted by treatment group into six pens (5.0 m×10.0 m) and fed concentrates (9.5 to 10.0 kg/d/head) and perennial ryegrass (1.3 kg/d/head). Concentrate was fed at approximately 1.5% of body weight (BW, as-fed basis) for the entire experimental period of 150 days. The ingredients and chemical composition of the concentrate and perennial ryegrass are presented in Table 1. Chemical composition of the experimental diets were analyzed by the methods of AOAC [8]. Commercial ethanol (30%, v/v, manufactured for human consumption by local distillery) was supplemented into the drinking water twice a day to meet final concentrations based on average water consumption (determined to 30 L/d/head after 2 weeks of pre-trial test) of finishing Hanwoo steers. Blood samples were obtained from jugular veins of experimental animals by using vacutainers before and after the experiment at 09:00 am prior to morning feeding, and serum was separated by centrifuging at 2,500×*g* for 10 minutes at 4°C and kept in a freezer (−80°C) until analysis. At the initiation (average 25.1 months of age) and termination (average 29.9 months of age) of the experiment, fat thickness and marbling score were measured in live animals to exclude genetic background of individual animals from the results and were obtained by using a portable ultrasonic instrument (Model R3, Samsung Medison Co., Ltd., Seongnam, Korea). The experiment was terminated when steers of each treatment group reached an age of 29.9±0.3 months.

### Carcass evaluation and samples

At termination, all experimental animals were transported to the local abattoir and slaughtered. After chilling the carcasses for 24 hours at 4°C, cold carcass weight, marbling degree, longissimus muscle (LM) area, fat thickness, meat color, fat color, texture, and maturity at the 13th rib were determined to obtain the animal’s grade for yield and quality according to the Korean Beef Carcass Grading Standard [9]. Five (5) gram liver samples from the right medial lobe and 5 g of sternocephalicus muscle were taken to measure TG levels in liver and muscle, respectively.

### Total lipid extraction

Samples of LM between the 12th and 13th ribs were removed from each carcass and transported at 4°C to the laboratory. After trimming the subcutaneous fat, the samples were homogenized (Polytron PT2100, Kinematica Inc., Bohemia, NY, USA) and stored in a freezer (−80°C) until analysis.

Total lipids were extracted from LM samples by a modified method of Folch et al [10]. Approximately 5 g of muscle tissue was homogenized with 5 mL of chloroform:methanol (2:1, v/v) and held at room temperature for 30 minutes to extract lipids. The homogenate was filtered through Whatman GF/C filters (Whatman Ltd., Maidstone, England) and rinsed with an additional 10 mL of chloroform:methanol. The extracted lipid was then combined with 8 mL of 0.74% KCl and vortexed for 1 min. After the phases were separated, the lipid layer was transferred into 20 mL scintillation vials, and the solvents were evaporated by heating at 60°C under nitrogen.

### Fatty acid composition

The extracted lipids from LM were acetylated by using the method of Lepage and Roy [11], and fatty acid composition was analyzed by using gas chromatography. Approximately 100 μL of lipid extract were converted to fatty acid methyl esters by adding 2 mL methanol:benzene (4:1, v/v) and 200 μL of acetyl chloride and heating for 40 minutes on a heating block. Isooctane (1 mL) and 6% potassium carbonate (8 mL) were added the mixture and centrifuged for 10 minutes at 1,500×*g*. The supernatant was used to analyze fatty acid composition by using a gas chromatograph (Clarus 500, Perkin Elmer, Shelton, CT, USA) equipped with a fused column SP 2560 (100 m length, 0.25 mm diameter) and nitrogen as the carrier gas (flow rate; 1 mL/min). The initial oven temperature was 200°C and the injector and detector temperatures were 220°C and 250°C, respectively.

### Melting point

The melting point of the lipid extracted from LM was measured by using a modified method of Chung et al [12]. The lipid was drawn into glass capillary tubes made for the measurement of the melting point. For each sample, 5 capillary tubes were made and frozen at −20°C. After freezing, the capillary tubes were suspended vertically in a chilled water bath with the portion of the tube containing the lipid submerged in the water. The water bath was heated at 2°C/min with constant stirring. The temperature of the water was monitored with a mercury thermometer and the melting point was determined as the temperature at which the lipid moved up the capillary tube and was recorded as the mean of five replicates for each sample.

### Serum metabolites and liver and muscle triglycerides

Serum glucose and TG concentrations were measured by using a kit obtained from Asan Pharm Co. (Seoul, Korea). NEFA concentrations were measured by using a commercial kit (Wako Co., Osaka, Japan). Plasma insulin was measured by using an ELISA kit for bovine insulin (Alpco, Salem, NH, USA).

The TG contents of the liver and muscle samples were determined by extracting total lipids with chloroform:methanol (2:1, v/v) as described by Folch et al [10]. Phospholipids were removed by separating the chloroform:methanol solution and the water phase following the method of Frayn and Maycock [13] which is a modified method of Denton and Randle [14]. The TG content was then quantified spectrophotometrically by using an enzymatic assay kit (Asan Pharm Co., Seoul, Korea).

### Statistical analysis

The least squares method was used to estimate ethanol effects on BW, average daily gain (ADG), carcass yield, and quality grades, fatty acid composition, melting point, serum metabolites and liver and muscle TG contents using linear model treating individual animal in the same treatment group as an independent variable using the formula:

$${\text{Y}}_{\text{ij}}=\mu +{\text{A}}_{\text{i}}+{\text{e}}_{\text{ij}}$$

where Y_ij_ = the observed value of ith treatment and jth individual, μ = overall mean, A_i_ = effect of treatment (i = 1, 2, 3), e_ij_ = assumed to be normally and independently distributed with mean zero and variances σ^2^.

The linear model was analyzed using SPSS (Release Ver. 19.0) [15]. The statistical significance of differences were tested at the 0.05% level and Turkey’s multiple range test was used for comparisons among treatment groups.

## RESULTS AND DISCUSSION

### Growth performances

There were no statistical differences in final BW or ADG of Hanwoo steers between control and ethanol-fed groups (Table 2).

Discovery programs in human medicine, especially those related to obesity and diabetes, have developed useful technologies involved in lipid and fatty acid metabolism [16–19]. In humans, chronic alcohol consumption perturbs lipid metabolism as it increases adipose tissue lipolysis and leads to ectopic fat deposition within the liver and the development of alcoholic fatty liver disease [20]. Ruminants (cattle, sheep, goats), however, possess a gastrointestinal tract anatomically and functionally different to those of humans and rodents, and, thereby, their lipid metabolism is also quite different [19]. In ruminants, adipose tissue is the principal site of *de novo* fatty acid synthesis [18,21].

Garrett and Meyer [6] reported that feeding denatured ethanol to Hereford steers produced no differences in ADG. However, Kreul et al [7] reported that when crossbred steers were fed diets with ethanol being supplemented as 0% (control), 2%, 4%, or 6% of dietary dry matter, their daily gains and feed efficiency were improved dose-dependently with an ADG being 36% greater (p<0.08) when ethanol comprised 6% of the ration’s dry matter. Higher energy levels in alcohol-containing diets may account partially for the improvement in feedlot performance. However, in the second part of the same report, when growing cattle were given *ad libitum* access to feed, cattle with different durations (0, 17, 24, 38, 66, and 122 days) of 5% ethanol treatment performed similarly. The addition of ethanol to beef cattle finishing rations increases the energy content of the diet; however, ethanol addition has no consistent effect on performance or carcass traits [7].

Li et al [22] mentioned that the addition of an adequate amount of alcohol to beef cattle can be effective in improving feed efficiency and meat quality. Alcohol, introduced in the rumen via the diet, is partially transformed to volatile fatty acids by microorganisms in the rumen [23], and feeding alcohol-fermented diet to lactating cows did not affect their dry matter intake [22]. Despite the fact that alcohol-consuming mice consumed more overall calories, their BW and percentage body fat were not significantly different from those of water-consuming mice [24], reportedly, because alcohol regulates insulin sensitivity without significantly affecting BW or body fat level [25]. In other reports, chronic alcohol decreased the weight of epididymal white adipose tissue [26,27] and subcutaneous white adipose tissue [28] in mice and muscle in humans [29].

Based on the previous results using small experimental animals and humans and the results obtained from the current study showing E-5 animals did not exhibit compensatory growth to overcome initial BW, it is considered that direct ethanol feeding to fattening steers through drinking water had no effects of growth performances.

### Carcass characteristics

There were no differences by feeding ethanol in carcass yield grade indices such as cold carcass weight, fat thickness, and LM area; however, the appearance frequencies of final yield grade for A (lean), B, and C (fatty) grades were 30%, 50%, and 20% for the control group, 20%, 50%, and 30% for the E-3 group, and 10%, 30%, and 60% for the E-5 group, respectively, showing a higher percentage of yield grade C by feeding ethanol for 5 months (Table 3). Although there were no statistical differences among group in yield grades, the overall numerical differences implied that alcohol supplementation could affect final yield grade.

Marbling score was not statistically different (p = 0.228) but numerically scored highly by feeding ethanol with the highest score observed in E-5 group followed by E-3 and control groups in the order. Similar to yield grade, numerical differences without statistical significances among treatment groups showed changes in final quality grades by feeding ethanol. The appearance frequencies of 1^++^ (the best), 1^+^, 1, and 2 grades were of 30%, 50%, 0%, and 20% for the control group, 10%, 80%, 10%, and 0% for the E-3 group, and 10%, 80%, 0%, and 10% for the E-5 group, respectively. There were no differences in meat color, fat color, texture, and maturity among the groups after feeding ethanol either 3 or 5 months.

In ultrasound results, increments of marbling scores after experiment comparing to the initiation were significantly (p< 0.05) higher in all groups while ethanol-fed animals showed numerically higher scores than the control animals (p = 0.735) (Table 4).

Garrett and Meyer [6] reported that by feeding alcohol in drinking water at a concentration of 240 mL of denatured ethanol per head daily to Hereford steers, the animals’ carcass fat level was slightly increased from 24.0% to 24.2% whereas carcass grades appearances for 6 animals in the control and denatured ethanol groups were, according to the USA standard, 4 “good” and 2 “choice” and 2 “good” and 4 “choice”, respectively. In the same report, the authors concluded that an ethyl alcohol supplement was not beneficial to lean meat production in cattle. Dressing percentage, ribeye area, percentages of kidney, heart, and pelvic fat, adjusted fat thickness, and marbling score were unaffected (p>0.05) regardless of dietary ethanol level or length of ethanol addition [7]. Unlike the results in the present study, which showed no significant change, Kreul et al [7] reported that the percentage of carcasses attaining the USA “choice” quality grade appeared to decrease linearly with increasing ethanol supplementation and concluded that, although not statistically different, the magnitude of the difference may have practical significance depending on how the cattle are marketed. In their second study, the short-term addition of ethanol appeared to increase the number of cattle reaching the choice quality grade, and the authors concluded that whether intramuscular lipid deposition is influenced by ethanol consumption was undetermined [7].

The TG content in the liver of the experimental animals were 2.08±0.28 μmol/g in control animals, 1.80±0.53 μmol/g in E-3 animals, and 1.48±0.55 μmol/g in E-5 animals, respectively, whereas the TG content in muscle were 3.54±0.32 μmol/g in control animals, 3.69±0.65 μmol/g in E-3 animals, and 3.56± 0.49 μmol/g in E-5 animals, respectively (Table 5). Three animals in the control and one animal in E-3 group were diagnosed with fatty liver by a veterinarian (data not shown). Kreul et al [7] reported that the combination of ethanol with a high concentrate ration did not affect the incidence of liver condemnation at slaughter.

The addition of an adequate amount of alcohol to beef cattle diets has been reported to be desirable as a means to improve feed efficiency and meat quality. Shin et al [30] observed a high alcohol concentration in steers fed alcohol-fermented feed (AFF), and it was speculated that there was an amount of alcohol absorption from the AAF diet into the blood via the rumen wall. Yan [31] found beneficial effects of AFF for improving the marbling score of Korean native steers, and Lin [32] observed that production of total volatile acids and propionate in the rumen were affected by supplementation of alcohol-added feed or AFF and may improve BW gain by Korean native steers via decreased protein degradation and increased fat synthesis [33]. Yan [31] observed that AFF had the beneficial effect of improving the marbling score of beef. However, a logical explanation for the improved meat quality associated with the provision of dietary alcohol has not been presented [33].

### Serum glucose, insulin, non-esterified fatty acid, and triglyceride

By feeding ethanol to finishing Hanwoo steers for 3 or 5 months (i.e., the E-3 or E-5 groups, respectively), serum glucose concentrations tended to decrease whereas insulin concentrations to increase (p>0.05). By feeding ethanol, NEFA concentrations tended to increase (p>0.510) whereas TG concentrations maintained consistent concentrations before and after the experiment (Table 6).

Serum glucose and TG concentrations obtained in the current study are in accordance with the others for finishing Hanwoo steers [34]. Previous reports [6,7] on feeding ethanol to beef cattle directly were focused on practical aspects, and therefore, it is difficult to find laboratory-based analytical data to explain changes in lipid metabolism in ruminants resulting from ethanol feeding. One study reported that feeding AFFs to Holstein lactating cows at 0%, 5%, 10%, and 15% of total mixed ration had no effect on blood concentrations of creatinine, total TG, glutamate oxaloacetate transaminase, and glutamate pyruvate transaminase [22].

### Fatty acid composition and melting point

Table 7 shows fatty acid profiles of LM of the experimental animals. The SFA comprised 40.78% to 41.75% of the total fatty acids. Among the SFA, myristic acid (C_14:0_) comprised 3.45% to 3.52%, palmitic acid (C_16:0_) 27.13% to 28.15%, and stearic acid (C_18:0_) 9.95% to 10.20% of the total fatty acids. The MUFA comprised 55.80% to 56.98% of total fatty acids, and among the MUFA, myristoleic acid (C_14:1_) comprised 1.25% to 1.49%, palmitoleic acid (C_16:1_) 5.66% to 5.93%, elaidic acid (C_18:1_) 1.36% to 1.45%, oleic acid (C_18:1n-9_) 47.31% to 48.27%, and vaccenic acid (C_18:1n7_) 0.22% to 0.23% of the total fatty acids. Oleic acid (C_18:1n-9_) comprised nearly 85% of the total MUFA. Polyunsaturated fatty acids formed 2.06% to 2.24% with linoleic acid (C_18:2_) 1.86% to 2.02% and linolenic acid (C_18:3_) 0.08%. Among the conjugated linoleic acids (C_18:2_), the cis-9, trans-11 isoform (CLA_9,11_) in the E-5 group was significantly (p = 0.043) higher than those in the control or E-3 groups, whereas no differences were found in the trans-10, cis-12 isoform (CLA_10,12_) (0.02% of the total fatty acids throughout the treatment groups). The ratio of unsaturated fatty acids (UFA) to SFA in the carcasses of experimental animals were ranged from 1.40 to 1.46 throughout the treatment groups, indicating a notably higher percentage of UFA than SFA in Hanwoo steers. There were no significant changes in the fatty acid composition of LM with regard to feeding ethanol to finishing Hanwoo steers except for the conjugated linoleic acid isoform CLA_9,11_ (Table 7).

A high proportion of MUFAs, including oleic acid, is reported to have benefits for human cardiovascular diseases [35, 36], blood pressure [37], and diabetes mellitus [38]. Besides these benefits of MUFAs, MUFAs in beef is also reported to have positive effects on palatability. Hanwoo beef with high proportions of MUFAs had high scores in palatability in panel tests comparing with imported beef having relatively low MUFA levels [39,40]. In addition, information provided by previous researchers implies that beef produced by a concentrate-based feeding system was highly rated for tenderness, juiciness, umami, and overall palatability.

The melting point of the lipids extracted from LM of Hanwoo steers in the current experiment ranged from 22.9°C to 23.9°C with no significant differences by feeding ethanol. It has been reported that a high proportion of oleic acid in beef significantly (p<0.05) decreases the melting point of the lipid extracted from sirloin of Hanwoo steers [5,39].

In conclusion, the results obtained in this study imply that feeding ethanol directly through drinking water of finishing Hanwoo steers caused lipolysis of existing body fats (probably abdominal fats), transported metabolites, glucose and NEFA, into serum, stimulated lipogenesis in intramuscular adipose tissues, increased marbling scores, and, as a result, improved final quality grades of the carcass. While most of the results in this study did not indicate statistical differences, the numerical values of the data were deemed important in practical applications to the efforts to improve intramuscular fat mass by feeding ethanol to Hanwoo steers. Regardless, further studies to determine changes in fat distributions to abdominal, subcutaneous, and intramuscular adipose tissues in beef carcasses and to analyze serum metabolites and hormones related to lipid metabolism in beef cattle are necessary.

## Figures and Tables

**Table 1:** Chemical compositions of concentrate and perennial ryegrass fed to finishing Korean cattle (Hanwoo) steers

Item	Concentrate	Perennial ryegrass
	------------------------ Formula, % -----------------------	
Corn grain	45.2	
Soybean bran	12.0	
Corn gluten feed	12.0	
Wheat bran	3.0	
Rapeseed meal	7.0	
Palm kernel meal	5.0	
Coconut meal	5.0	
Distillers dried grain	4.0	
Cane molasses	5.0	
Lime	1.0	
Sodium bicarbonate	0.5	
Vitamin mix	0.1	
Mineral mix	0.1	
Mineral oil	0.1	
Total	100.0	
	---------------- Chemical composition, % --------------	
Dry matter	87.15	92.38
Crude protein	12.83	7.74
Ether extract	4.21	1.17
Crude fiber	8.47	33.79
Crude ash	5.15	5.18
NFE	56.49	44.5
Ca	0.75	0.25
P	0.43	0.16
ADF	12.66	40.15
NDF	25.31	63.86
TDN	74.00	48.60

NFE, nitrogen-free extract; ADF, acid detergent fiber; NDF, neutral detergent fiber; TDN, total digestible nutrients (calculated).

**Table 2:** Effects of feeding ethanol on growth performances of finishing Korean cattle (Hanwoo) steers

Items	Control1)	E-32)	E-53)	p value
No. of steers	10	10	10	-
Initiation age, months	25.2±0.44)	25.0±0.3	25.0±0.3	-
Termination age, months	29.9±0.4	29.7±0.3	30.0±0.3	-
Body weight (kg)				
Initial	656.2±38.9	680.4±53.2	643.6±48.9	0.229
Final	744.2±53.8	769.0±60.4	725.4±54.4	0.239
Total gain	88.0±20.31	88.6±26.49	81.8±20.73	0.761
ADG	0.61±0.14	0.61±0.19	0.56±0.14	0.758
Feed intake (DM, kg/head/d)5)				
Concentrate	8.28	8.72	8.70	-
Perennial ryegrass	1.20	1.20	1.20	-
Total	9.48	9.92	9.90	-
Feed conversion ratio	15.54	16.26	17.68	-

ADG, average daily gain; DM, dry matter.

1) Commercial finishing concentrate.

2) Commercial finishing concentrate+ethanol [1.44% (v/v) of ethanol in drinking water for 3 months].

3) Commercial finishing concentrate+ethanol for 5 months [0.72% (v/v) for two months followed by 1.44% for 3 months].

4) Mean±standard deviation

5) Means of group-feeding.

**Table 3:** Effects of feeding ethanol on carcass characteristics of finishing Korean cattle (Hanwoo) steers

Items	Control1)	E-32)	E-53)	p-value
Cold carcass wt. (kg)	435.7±34.14)	455.5±38.6	425.3±38.8	0.205
Yield grade				
Fat thickness (mm)	13.1±4.8	12.6±3.3	14.3±3.9	0.161
Loin area (cm^2^)	98.1±9.9	94.1±8.5	89.8±15.9	0.309
Yield index	65.5±3.3	64.9±2.1	62.6±4.0	0.116
Appearance ratio5)				
A	3 (30.0)6)	2 (20.0)	1 (10.0)	-
B	5 (50.0)	5 (50.0)	3 (30.0)	-
C	2 (20.0)	3 (30.0)	6 (60.0)	-
Quality grade;				
Marbling score7)	6.0±2.3	6.3±1.1	6.7±0.9	0.228
Meat color8)	5.0±0.6	5.0±0.0	5.0±0.0	0.381
Fat color9)	3.0±0.0	3.1±0.0	2.9±0.3	0.381
Texture10)	1.0±0.3	1.1±0.0	1.1±0.3	0.612
Maturity11)	2.0±0.0	2.0±0.0	2.0±0.0	-
Appearance ratio12)				
1++	3 (30.0)6)	1 (10.0)	1 (10.0)	-
1+	5 (50.0)	8 (80.0)	8 (80.0)	-
1	-	1 (10.0)	-	-
2	2 (20.0)	-	1 (10.0)	-

1) Commercial finishing concentrate,

2) Commercial finishing concentrate+ethanol [1.44% (v/v) of ethanol in drinking water for 3 months].

3) Commercial finishing concentrate+ethanol for 5 months [0.72% (v/v) for two months followed by 1.44% for 3 months].

4) Mean±standard deviation.

5) Final yield grade: A (lean), B (medium), C (fatty).

6) Number in parenthesis means percentages.

7) 9 = the most abundant, 1 = devoid.

8) 1 = bright red, 7 = dark red.

9) 1 = white, 7 = yellowish.

10) 1 = fine, 3 = coarse.

11) 1 = immature, 9 = mature.

12) Final quality grade: 1^++^ (the best), 1^+^, 1, 2, 3 (the worst).

**Table 4:** Fat thickness and marbling score of finishing Korean cattle (Hanwoo) steers measured by ultrasound scanner before and after feeding ethanol

Items	Control1)	E-32)	E-53)	p-value
Fat thickness (mm)				
Before	9.2±2.44)	8.4±2.1	10.3±2.6	0.209
After	11.1±2.9	10.6±2.4	12.8±3.0	0.209
p-value	0.470	0.441	0.928	-
Marbling score				
Before	3.0±0.7	3.1±0.3	3.5±0.5	0.096
After	4.3±1.3*	4.7±0.8*	4.5±1.3*	0.735
p-value	0.020	0.002	0.002	-

1) Commercial finishing concentrate.

2) Commercial finishing concentrate+ethanol [1.44% (v/v) of ethanol in drinking water for 3 months].

3) Commercial finishing concentrate+ethanol for 5 months [0.72% (v/v) for two months followed by 1.44% for 3 months].

4) Mean±standard deviation.

* Indicates statistical differences between before and after experiment.

**Table 5:** Effects of feeding ethanol on triglyceride contents of liver and sternocephalicus muscle of finishing Korean cattle (Hanwoo) steers

Items	Control1)	E-32)	E-53)	p value
---------------- Triglyceride (μmol/g) ---------------				
Liver	2.08±0.784)	1.80±0.53	1.48±0.55	0.129
Muscle	3.54±0.32	3.69±0.65	3.56±0.49	0.780

1) Commercial finishing concentrate.

2) Commercial finishing concentrate+ethanol [1.44% (v/v) of ethanol in drinking water for 3 months].

3) Commercial finishing concentrate+ethanol for 5 months [0.72% (v/v) for two months followed by 1.44% for 3 months].

4) Mean±standard deviation.

**Table 6:** Effects of feeding ethanol on serum glucose, insulin, non-esterified fatty acid, and triglyceride content of finishing Korean cattle (Hanwoo) steers

Items		Control1)	E-32)	E-53)	p-value
Glucose (mg/dL)	Before	76.42±4.004)	83.11±6.53	82.49±5.30	0.018
	After	73.87±12.62	74.66±6.02	76.55±9.02	0.816
	p-value	0.437	0.282	0.516	-
Insulin (ng/mL)	Before	3.08±1.55	3.01±1.65	2.75±1.39	0.885
	After	3.04±1.39	3.56±0.85	3.27±1.31	0.654
	p-value	0.094	0.950	0.712	-
NEFA (mEq/L)	Before	0.48±0.05	0.47±0.06	0.47±0.12	0.950
	After	0.43±0.12	0.53±0.12	0.51±0.10	0.090
	p-value	0.444	0.642	0.510	-
TG (mg/mL)	Before	0.16±0.04	0.16±0.05	0.17±0.05	0.754
	After	0.14±0.05	0.16±0.04	0.17±0.04	0.428
	p-value	0.196	0.203	0.077	-

NEFA, non-esterified fatty acid; TG, triglyceride.

1) Commercial finishing concentrate.

2) Commercial finishing concentrate+ethanol [1.44% (v/v) of ethanol in drinking water for 3 months].

3) Commercial finishing concentrate+ethanol for 5 months [0.72% (v/v) for two months followed by 1.44% for 3 months].

4) Mean±standard deviation.

**Table 7:** Effects of feeding ethanol on fatty acid composition and melting point of loin muscle of finishing Korean cattle (Hanwoo) steers

Fatty acid (%)	Control1)	E-32)	E-53)	p-value
Myristic acid (C_14:0_)	3.45±0.454)	3.52±0.53	3.49±0.51	0.943
Myristoleic acid (C_14:1_)	1.27±0.58	1.49±0.32	1.25±0.48	0.486
Palmitic acid (C_16:0_)	27.13±1.66	27.93±1.96	28.15±1.46	0.383
Palmitoleic acid (C_16:1_)	5.77±0.84	5.93±0.70	5.66±0.75	0.734
Stearic acid (C_18:0_)	10.20±2.05	9.95±1.16	10.11±1.08	0.928
Elaidic acid (C_18:1_)	1.45±0.37	1.40±0.22	1.36±0.24	0.784
Oleic acid (C_18:1n-9_)	48.27±2.19	47.50±3.05	47.31±1.87	0.644
Vaccenic acid (C_18:1n7_)	0.22±0.04	0.23±0.03	0.22±0.03	0.698
Linoleic acid (C_18:2_)	2.02±0.50	1.86±0.32	2.21±1.04	0.521
Linolenic acid, (C_18:3_)	0.08±0.01	0.08±0.02	0.08±0.01	0.354
CLA_9,11_ (C_18:2,9-_*_cis_*_,11-_*_trans_*)	0.12±0.02^ab^	0.10±0.02^b^	0.14±0.05^a^	0.043
CLA_10,12_ (C_18:2,10-_*_trans_*_,12-_*_cis_*)	0.02±0.01	0.02±0.01	0.02±0.01	0.831
SFA	40.78±2.46	41.40±3.29	41.75±2.20	0.718
UFA	59.22±2.46	58.60±3.29	58.25±2.20	0.718
MUFA	56.98±2.88	56.54±3.25	55.80±1.77	0.621
PUFA	2.24±0.51	2.06±0.33	2.45±1.03	0.459
U/S, ratio	1.46±0.14	1.43±0.19	1.40±0.13	0.708
M/S, ratio	1.41±0.15	1.38±0.19	1.34±0.11	0.653
Melting point (°C)	23.9±3.8	23.7±3.7	22.9±3.6	0.813

SFA, saturated fatty acid; UFA, unsaturated fatty acid; MUFA, monounsaturated fatty acid; PUFA, polyunsaturated fatty acid; U/S, UFA/SFA; M/S, MUFA/SFA.

1) Commercial finishing concentrate.

2) Commercial finishing concentrate+ethanol [1.44% (v/v) of ethanol in drinking water for 3 months].

3) Commercial finishing concentrate+ethanol for 5 months [0.72% (v/v) for two months followed by 1.44% for 3 months].

4) Mean±standard deviation.

ab Means in the same row with different superscripts are significantly different.
